# Wunderlich Syndrome in a Patient With Coexisting Giant Angiomyolipoma and Multicystic Clear Cell Renal Cell Carcinoma: A Case Report

**DOI:** 10.7759/cureus.109485

**Published:** 2026-05-23

**Authors:** Rahul Gupta, Prince Rathi, Nalini Bansal, Jyoti Koli

**Affiliations:** 1 Gastrointestinal Surgery, Synergy Institute of Medical Sciences, Dehradun, IND; 2 Surgery, Synergy Institute of Medical Sciences, Dehradun, IND; 3 Histopathology, Institute of Gastro and Hepatopathology, Gurugram, IND; 4 Radiation Oncology, Synergy Institute of Medical Sciences, Dehradun, IND

**Keywords:** clear cell renal carcinoma, intra-tumoral hemorrhage, renal angiomyolipoma, spontaneous hemoperitoneum, unilateral nephrectomy, wunderlich syndrome

## Abstract

Angiomyolipoma (AML) is a benign mesenchymal renal neoplasm composed of dysmorphic blood vessels, smooth muscle cells, and adipose tissue. Although most AMLs are asymptomatic and incidentally detected, large tumors may present with spontaneous hemorrhage resulting in Wunderlich syndrome (WS), a rare urological emergency characterized by spontaneous nontraumatic renal bleeding. Renal cell carcinoma (RCC), on the other hand, is the most common malignant epithelial tumor of the kidney. The synchronous occurrence of AML and RCC within the same kidney is rare. We report the case of a 64-year-old woman who presented with acute left flank pain, hypotension, anemia, and a palpable abdominal mass. Imaging revealed a giant hemorrhagic AML arising from the upper pole of the left kidney with associated hemoperitoneum. Following resuscitation, the patient underwent emergency radical left nephrectomy. Histopathological examination demonstrated a ruptured giant AML along with an incidentally detected multicystic clear cell RCC. Immunohistochemistry confirmed the diagnoses with HMB45 positivity in AML and CD10, CK7, and PAX8 positivity in RCC. This case highlights the rare coexistence of AML and RCC in the same kidney and emphasizes the importance of careful pathological evaluation in patients presenting with hemorrhagic renal masses. Early recognition and prompt surgical intervention are essential for favorable outcomes in WS.

## Introduction

Renal angiomyolipoma (AML) is a benign mesenchymal neoplasm accounting for approximately 0.3-0.6% of all renal tumors [1]. Histologically, AML is composed of variable proportions of mature adipose tissue, smooth muscle cells, and thick-walled blood vessels. Most AMLs occur sporadically; however, some are associated with tuberous sclerosis complex (TSC). Small AMLs are usually asymptomatic and detected incidentally, whereas larger tumors may present with abdominal pain, hematuria, or life-threatening hemorrhage.

Wunderlich syndrome (WS) refers to spontaneous nontraumatic hemorrhage into the subcapsular and perirenal spaces. It is an uncommon but potentially fatal condition, most frequently associated with renal neoplasms such as AML and renal cell carcinoma (RCC) [2]. Clinically, WS is characterized by Lenk’s triad consisting of acute flank pain, a palpable flank mass, and hypovolemic shock.

RCC is the most common malignant epithelial tumor of the kidney, with clear cell RCC representing the predominant histological subtype. The coexistence of AML and RCC within the same kidney is exceedingly rare, with several dozen cases reported in the literature [3,4]. Moreover, development of spontaneous hemorrhage leading to acute presentations and deranged renal functions can significantly compromise visualization of the coexisting lesions. We present a rare case of ruptured giant AML causing WS with synchronous multicystic clear cell RCC in the ipsilateral kidney.

## Case presentation

A 64-year-old woman with a history of diabetes mellitus presented to the emergency department with severe left flank pain, nausea, vomiting, and oliguria of one-day duration. On examination, the patient appeared pale and tachycardic, with hypotension suggestive of hemodynamic instability. Abdominal examination revealed a large, ill-defined, tender mass palpable in the left flank. There was no abdominal guarding or rigidity.

Laboratory investigations demonstrated severe anemia with hemoglobin of 5.3 g/dL, leukocytosis with a total leukocyte count of 16,160/cu.mm, hypoalbuminemia with serum albumin of 2.4 g/dL, and deranged renal function tests with serum creatinine of 1.8 mg/dL.

Due to deranged renal functions, non-contrast computed tomography (CT) of the abdomen and pelvis was performed. It demonstrated a large heterogeneous exophytic mass arising from the upper pole of the left kidney measuring 16.4 × 10 × 12 cm. The lesion showed predominant fat attenuation with multiple hyperdense areas suggestive of intratumoral hemorrhage. Mild-to-moderate hemoperitoneum and cholelithiasis were also noted. Based on the radiological findings, a provisional diagnosis of giant renal AML with internal hemorrhage resulting in WS was made. As the patient was in hemorrhagic shock with deranged renal functions, no further imaging or selective arterial embolization could be performed, and the patient was planned for emergency laparotomy.

Following aggressive fluid resuscitation and blood transfusion, the patient underwent emergency exploratory laparotomy. Intraoperatively, moderate hemoperitoneum was encountered. A large retroperitoneal mass measuring approximately 20 × 14 × 10 cm was identified arising from the upper pole of the left kidney, with rupture into the peritoneal cavity through the left mesocolon. A left radical nephrectomy was performed after ligation and division of the renal vessels. Hemostasis was secured, and a drain was placed. The operative time was 165 minutes, and the estimated blood loss was approximately 1 liter. The postoperative course was uneventful, and the patient was discharged on postoperative day six.

**Figure 1:**
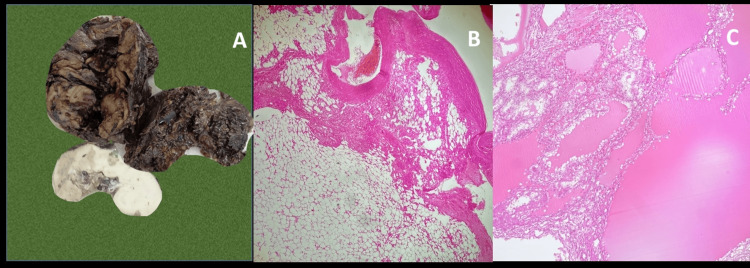
(A) Gross specimen showing a large hemorrhagic renal mass with areas of necrosis and yellowish-tan tissue. (B) Histopathological examination showing mature adipose tissue, spindle-shaped smooth muscle cells, and dysmorphic blood vessels consistent with angiomyolipoma (H&E, ×10). (C) Microscopy of the multicystic lesion showing cystic spaces lined by clear cells consistent with multicystic clear cell renal cell carcinoma (H&E, ×10).

Gross examination of the nephrectomy specimen revealed a heterogeneous mass with areas of hemorrhage, necrosis, and yellowish-tan tissue arising from the upper pole of the kidney (Figure 1A). In addition, a small multicystic lesion was identified in the renal cortex of the mid-pole region. Microscopic examination of the large mass demonstrated the characteristic triphasic pattern of AML consisting of mature adipose tissue, spindle-shaped smooth muscle cells, and thick-walled dysmorphic blood vessels (Figure 1B). Histopathological examination of the multicystic lesion showed multiple cystic spaces lined by clear cells consistent with multicystic clear cell RCC (Figure 1C). The tumor was confined to the kidney with an intact capsule.

**Figure 2:**
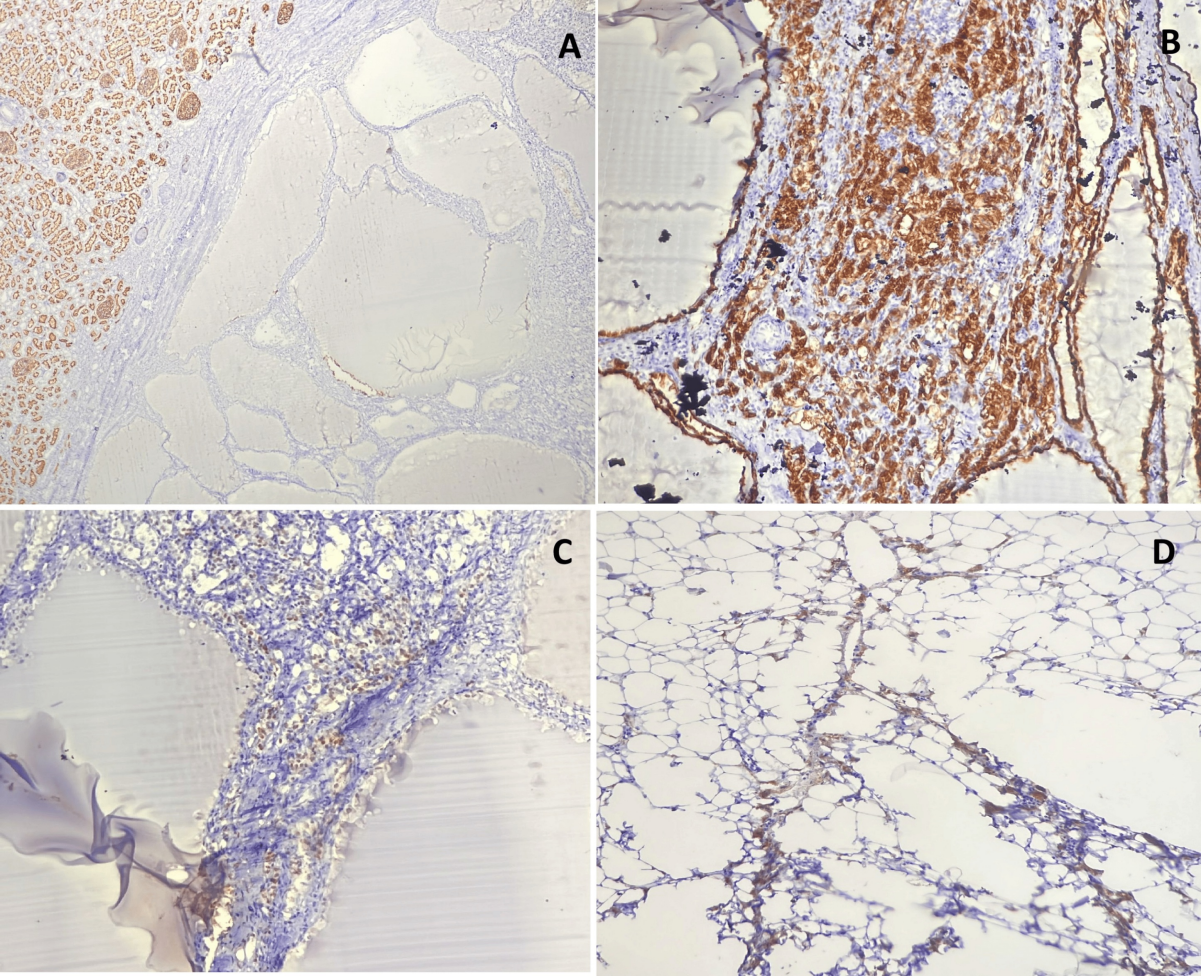
Immunohistochemistry demonstrating positivity for CD10 (A), CK7 (B), and PAX8 (C) in the renal cell carcinoma component and HMB45 positivity (D) in the angiomyolipoma component.

Immunohistochemical analysis revealed HMB45 positivity in the AML component, while the RCC component showed positivity for CD10, CK7, and PAX8 (Figure 2). The final diagnosis was ruptured giant AML with synchronous multicystic clear cell RCC (pathological stage pT1).

At one-year follow-up, the patient remained asymptomatic with no evidence of recurrence.

## Discussion

AML is a benign renal neoplasm that predominantly affects middle-aged women and may occur sporadically or in association with TSC [1]. Although most AMLs are asymptomatic, larger lesions are associated with an increased risk of spontaneous hemorrhage due to the presence of abnormal aneurysmal blood vessels lacking elastic tissue. Tumors larger than 4 cm are particularly prone to bleeding.

WS is a rare clinical entity defined as spontaneous nontraumatic renal hemorrhage into the subcapsular and perirenal spaces. AML is among the most common causes of WS, followed by RCC and vascular disorders [2,5]. Patients typically present with Lenk’s triad of acute flank pain, flank mass, and hypovolemic shock, all of which were present in our patient.

Contrast-enhanced CT is considered the imaging modality of choice for diagnosing renal hemorrhage and identifying the underlying etiology. However, in the present case, only non-contrast CT could be performed because of impaired renal function. Consequently, the small RCC lesion remained undetected preoperatively. Although contrast-enhanced CT is the preferred modality for evaluating renal hemorrhage, impaired renal function limited imaging to non-contrast CT in this case. Situations like these-where acute clinical conditions restrict optimal imaging-highlight how easily small lesions may be missed before surgery. Recent literature in the urologic oncology suggests that relying on a single imaging method can be challenging in complex presentations, underscoring the need for comprehensive assessment when evaluating hemorrhagic renal masses [6].

The coexistence of AML and renal cell carcinoma within the same kidney is uncommon and is most frequently associated with TSC, although sporadic cases have also been documented [3,4]. The pathogenesis underlying the coexistence of these tumors remains unclear. These lesions may occur as collision tumors, within the same mass, or as separate synchronous lesions within the same or contralateral kidney.

Collision tumors are defined as the presence of multiple non-confluent tumors of histologically different types in the same part of the kidney [7]. Composite tumors consist of distinct pathological tumor types within the same mass, often representing divergent differentiation of a single neoplasm [7]. By contrast, synchronous tumors refer to the presence of two different tumors simultaneously or within a short time interval, in separate regions of the same or contralateral kidney [7]. Therefore, based on the above definitions, they were identified as synchronous renal tumors in the index case.

Management of AML depends on tumor size, symptoms, and hemodynamic stability [8]. Selective arterial embolization may be considered in hemodynamically stable patients with bleeding AML. However, emergency nephrectomy remains the treatment of choice in patients with massive hemorrhage, hemodynamic instability, or suspicion of malignancy. In our patient, radical nephrectomy provided definitive management for both hemorrhagic AML and incidental RCC.

Histopathological evaluation and immunohistochemistry are essential for definitive diagnosis. AML typically demonstrates positivity for melanocytic markers such as HMB45, while RCC expresses epithelial markers including CD10, CK7, and PAX8.

There are no established follow-up protocols for synchronous renal tumors. Patients with TSC require lifelong surveillance with abdominal CT or magnetic resonance imaging every one to three years [9]. In sporadic cases, follow-up depends on the patient’s initial tumor stage, grade, and surgical approach for RCC [10]. Typically, after radical nephrectomy, annual clinical examination with renal function assessment for 3 to 5 years is required. In addition, cross-sectional imaging must be performed every six to 12 months to look for recurrence or new lesions in the contralateral kidney [10].

## Conclusions

The coexistence of giant renal AML and multicystic clear cell RCC within the same kidney is exceptionally rare. Giant AML may present with life-threatening spontaneous hemorrhage resulting in WS. Careful pathological examination of nephrectomy specimens is essential because associated malignancies may remain undetected on imaging, particularly in emergency settings such as acute hemorrhage. Prompt surgical intervention can be lifesaving and may achieve favorable oncological outcomes.
